# Awareness, Attitude, and Acceptability of Healthcare Workers About COVID-19 Vaccination in Western India

**DOI:** 10.7759/cureus.18400

**Published:** 2021-09-30

**Authors:** Sanjeeta Dara, Suresh K Sharma, Ashok Kumar, Akhil D Goel, Vidhi Jain, Mukesh C Sharma, Manoj K Gupta, Suman Saurabh, Pankaj Bhardwaj, Sanjeev Misra

**Affiliations:** 1 College of Nursing, All India Institute of Medical Sciences, Jodhpur, IND; 2 Community Medicine & Family Medicine, All India Institute of Medical Sciences, Jodhpur, IND; 3 Microbiology, All India Institute of Medical Sciences, Jodhpur, IND; 4 Community Medicine & Family Medicine and School of Public Health, All India Institute of Medical Sciences, Jodhpur, IND; 5 Surgical Oncology, All India Institute of Medical Sciences, Jodhpur, IND

**Keywords:** health care workers, vaccine hesitancy, vaccine acceptance, immunization, sars-cov-2

## Abstract

Introduction

In the coronavirus disease 2019 (COVID-19) pandemic, healthcare workers (HCWs) are at the frontline around the world and categorized as a priority group for COVID-19 vaccines. Our study aimed to find out the COVID-19 vaccine awareness, attitude, and acceptance in HCWs in western India.

Methods

A cross-sectional study was carried out between January 14 and January 28, 2021, at a tertiary care hospital located in western India. Data were collected anonymously using Google Forms. Descriptive statistics were used to determine the sociodemographic variables. The knowledge and attitude of HCWs were analyzed using mean and SD. Multivariate analysis was done to find out the association between participants’ attitudes with demographic characteristics.

Results

Of the total health care workers, 498 answered the survey. The mean age of participants was 29.8 years (SD 6.4), and 354 (71.1%) were male. Among the respondents, 445 (89.4%) would accept a COVID-19 vaccine when available. Four-hundred seventy-six (476) HCWs (95.6%) had excellent knowledge regarding COVID-19 and COVID-19-appropriate behavior. The majority of the subjects (399) had a neutral attitude toward COVID-19 vaccination. Health care professionals (doctors and nurses) had higher acceptance for vaccination against COVID-19 than non-professionals.

Conclusions

The higher rates of COVID-19 vaccine acceptability and the excellent knowledge among HCWs will directly enhance the level and acceptability of vaccine among the general population and will definitely help in reducing the mortality and morbidity related to COVID-19.

## Introduction

Vaccination is a very effective approach to reduce morbidity and mortality among the population [1-2]. In public health, vaccination is one of the most important advances to control communicable diseases [3]. It was possible to eradicate smallpox only with vaccination [4]. The novel coronavirus disease 2019 (COVID-19) pandemic, caused by severe acute respiratory syndrome coronavirus 2 (SARS-CoV-2), was declared a "public health emergency of international concern" by WHO on January 30, 2020, and a “global pandemic” on March 11, 2020 [5]. SARS-CoV-2 is transmitted through close contact and droplet and aerosol transmission, therefore, proper infection control practices need to be followed. The control of COVID-19 infections can be achieved by vaccination of the population or natural infection along with the constant wearing of masks, hand washing, and social distancing [6-7]. However, the results of the natural infection are dangerous and a large number of the human population need to be infected with the virus. The development of herd immunity by mass vaccination has been a very successful and powerful strategy for halting the spread of many communicable diseases. Therefore, vaccination appears one of the most promising measures to control the spread of COVID-19 [8-9].

Vaccination has made the greatest contribution to global human health and herd immunity is considered as the main concept for epidemic control [10]. The development of a new vaccine has been a long process that at least takes 10 to 15 years. The mumps vaccine was the fastest developed vaccine and the same was also approved for use, which took almost five years. Hence, it was clearly a challenge to develop a vaccine against COVID-19 in a very short span. The development of COVID-19 vaccines is a pivotal challenge for scientists. Now many vaccines have been approved for their emergency use and many are in phase three trials and some of them were pre-clinical testing. After the clinical development of the COVID-19 vaccine, the next challenge is the distribution and acceptance of the COVID-19 vaccine in health care workers and the general population [11]. 

Healthcare workers are at the highest risk during this pandemic, as they are in direct contact with patients in COVID-19 designated care areas and are at risk of exposure to SARS-CoV-2. Besides this, these health care workers can be the source of infection to their families, patients, and relatives. Healthcare workers, senior citizens, and people with co-morbidities and chronic illnesses are at greatest risk due to COVID-19 infections and priority group for getting a vaccination against COVID-19 too [12]. High rates of acceptance and coverage of vaccination by its beneficiaries result in the success of an immunization program. Healthcare workers are a major and reliable source of information for vaccines that’s why HCWs can act as role models for the general population for getting a vaccination against COVID-19. It is important to consider healthcare workers' acceptability, knowledge, awareness, and attitudes about COVID-19 vaccination [13]. Health care professionals have a pivotal role in maintaining public trust in vaccination. That’s why the current study aimed to assess the acceptability, awareness, and attitudes about COVID-19 vaccination and COVID-19-appropriate behavior adapted by health care workers.

## Materials and methods

Design

A cross-sectional study was conducted between January 14 and January 28, 2021, to assess the awareness, acceptability, and attitude of healthcare workers about COVID-19 vaccination at a tertiary level hospital located in western India. This study was approved by the institutional ethical committee (AIIMS/IEC/2021/3255), All India Institute of Medical Sciences, Jodhpur, Rajasthan (India). 

Sample

Convenience sampling was utilized for all HCWs in a selected tertiary care hospital, All India Institute of Medical Sciences, Jodhpur, Rajasthan (India). The sample size was calculated assuming the expected proportion of awareness/attitude as 50%. We estimated a sample size of 423 at 10% relative precision, 95% CI, and 10% contingency. Considering a 5% non-response rate, we included a total of 450 participants in the present study [14].

Tool

An online questionnaire was created using Google Forms and converted into the local language (Hindi) by a language translator; internal reliability was checked for the same later. The questionnaire included three parts: (i) demographic information, including age, gender, occupation, qualification, COVID-19 positive status, any chronic illnesses, and willingness to receive COVID-19 vaccination; (ii) knowledge of anti-SARS-CoV-2 was assessed by a self-structured tool, which was validated by various experts and reliability was also checked through a pilot study;(iii) attitude toward SARS-CoV-2 vaccines was assessed by a standardized Vaccination Attitudes Examination (VAX) scale developed by Leslie R. Martin and Keith J. Petrie [15-16]. Convergent validity and internal reliability (Cronbach’s alphas = 0.77-0.93) were established. An additional question with an open-ended answer was provided for those who declared that they did not want to be vaccinated, exploring the reasons for personal vaccine hesitancy. The survey tool was distributed through emails and social media platforms in various health care workers' official groups of the institute. The data were collected anonymously and their mail IDs were collected to prevent duplication. We received a total of 498 responses and all were included in the final analysis.

Statistical analysis

The data were analyzed using Statistical Package for Social Sciences (SPSS) version 23 (IBM Corp, Armonk, NY) and were analyzed using mean and standard deviation. Categorical variables were presented by using frequency and percentage. Descriptive statistics were used to determine the sociodemographic variables. The knowledge and attitude of HCWs were analyzed with mean and SD. A multi-variate analysis of participants’ attitudes with demographic characteristics was also done.

## Results

The mean age of participants was of 29.8 years (SD 6.40) and 354 (71.1%) subjects were male. A total of 445 (89.4%) were ready to be vaccinated against COVID-19 when it would become available. Those who didn’t want to receive the vaccine had mainly safety issues, were concerned about the side effects of the vaccine, had chronic illnesses, or were pregnant or breastfeeding mothers. Table 1 summarizes the demographic characteristics of study participants.

**Table 1:** Demographic details of study participants (N=498)

Demographic Characteristics	Categories	Frequency (%)
Age (in years)	≤25	123 (24.7)
	>25	375 (75.3)
Gender	Male	354 (71.1)
	Female	144 (28.9)
Occupation	Professionals	294 (59.0)
	Non-professionals	204 (41.0)
Qualification	Post-graduate	271 (54.4)
	Graduate	130 (26.1)
	Secondary	05 (1.0)
	Preliminary	92 (18.5)
Have you ever tested positive for COVID-19?	Yes	130 (26.1)
	No	368 (73.9)
Have any of your family members tested positive for COVID-19?	Yes	78 (15.7)
	No	420 (84.3)
Do you suffer from any chronic illness?	Yes	28 (5.6)
	No	470 (94.4)
Are you willing to receive the COVID-19 vaccine?	Yes	445 (89.4)
	No	53 (10.6)

Four-hundred ninety-six respondents (99.6%) knew that SARS-CoV-2 is a virus and 472 (94.8%) were aware of the availability of a vaccine against COVID-19. The majority (97.2%) of study participants knew that India is making its own vaccine and almost half of the respondents (n=264, 53%) had a misconception that the Government of India made it mandatory for all to receive the vaccine (Table 2).

**Table 2:** Frequency and percentage of participants’ COVID-19 knowledge (N=498) PPE: personal protective equipment

Knowledge Statements	Responses Frequency (%)	
	Yes	No
Is SARS-CoV-2, which causes COVID-19, a virus?	496 (99.6)	2 (0.4)
Is there any vaccine available for COVID-19?	472 (94.8)	26 (5.2)
Is India making its own COVID-19 vaccine?	484 (97.2)	14 (2.8)
Is vaccination mandatory for all in India?	264 (53.0)	234 (47.0)
Will you wear a mask after getting the COVID-19 vaccine?	472 (94.8)	26 (5.2)
Will you follow hand hygiene practices after getting vaccinated for COVID-19?	481 (96.6)	17 (3.4)
Will you maintain social distancing after getting vaccinated for COVID-19?	475 (95.4)	23 (4.6)
Will you wear full PPE after getting vaccinated for COVID-19 in COVID wards?	428 (85.9)	70 (14.1)
If offered, would you prefer an Indian vaccine?	386 (77.5)	112 (22.4)

The majority of subjects (95.6%) had excellent knowledge regarding COVID-19 and COVID-19-appropriate behavior, as evident by the mean score of 7.17 ± SD 0.95, as shown in Table 3.

**Table 3:** Frequency, percentage, mean, and SD of COVID-19 knowledge level (N=498)

Level of knowledge	Score	Frequency	Percentage	Mean	Standard deviation
Poor knowledge	1-3	5	1%		
Good knowledge	4-5	17	3.4%	7.17	0.95
Excellent knowledge	6-8	476	95.6%		

Two-hundred twenty-nine (46%) study subjects had a negative attitude toward vaccine safety, yet 303 (68.8%) subjects feel protected from COVID-19 infection in the future after getting the COVID-19 vaccine. Thirty-eight point two percent (38.2%) of study subjects were worried about the unknown side effects of the vaccine. One-hundred ninety-three (39.2) subjects preferred natural immunity as compared to vaccination but they had a neutral attitude to whether natural exposure to viruses gives the safest protection against COVID-19 or being exposed to COVID-19 naturally is safer for the immune system than being exposed through vaccination (Table 4).

**Table 4:** Frequency and percentage of participants’ attitude toward COVID-19 vaccination (N=498)

Attitude Statements	Responses Frequency (%)		
	Strongly Disagree	Neutral	Strongly Agree
I feel that the COVID-19 vaccine is very safe	229 (46.0)	209 (42.0)	60 (12.0)
I can rely on the COVID-19 vaccine to prevent serious infection with COVID- 19.	211 (42.4)	218 (43.8)	69 (13.9)
I feel fully protected from COVID-19 infections in the future after getting the COVID-19 vaccine.	02 (0.4)	193 (38.8)	303 (68.8)
Although the COVID-19 vaccine appears to be safe, there may be problems that we haven’t yet discovered.	60 (12.0)	228 (45.8)	210 (42.2)
COVID-19 vaccine can cause unforeseen problems in individuals	121 (24.3)	187 (37.6)	190 (38.2)
I worry about the unknown effects of the COVID-19 vaccine in the future.	105 (21.1)	215 (43.2)	178 (35.7)
COVID-19 vaccine will make a lot of money for pharmaceutical companies but will not bring much benefit to common people.	155 (31.1)	226 (45.4)	117 (23.5)
Authorities promote the COVID-19 vaccine for financial gain, not for people’s health.	211 (42.4)	227 (45.6)	60 (12.0)
COVID-19 vaccination programs are a fraud.	286 (57.4)	178 (35.7)	34 (6.8)
Natural immunity will last longer than the COVID-19 vaccination.	114 (22.9)	189 (38.0)	195 (39.2)
Natural exposure to viruses gives the safest protection against COVID-19.	132 (26.5)	203 (40.8)	163 (32.7)
Being exposed to COVID-19 naturally is safer for the immune system than being exposed through vaccination.	135 (27.1)	214 (43.0)	149 (29.9)

The majority of subjects (399) had a neutral attitude toward COVID-19 vaccination as evident by the mean score of 40.04 ± SD 7.97 as shown in Table 5.

**Table 5:** Frequency, percentage, mean, and SD of COVID-19 vaccination attitude

Level of the knowledge	Score	Frequency	Percentage	Mean	Standard deviation
Non-favorable attitude	12-32	49	9.8%		
Neutral	32-52	399	80.1%	40.04	7.97
Favorable attitude	52-72	50	10%		

HCWs that were willing to receive the COVID-19 vaccine and their attitude toward the safety of COVID-19 vaccinations have a significant influence/association as assessed by participants’ attitude association with demographic characteristics in the multivariate analysis (Table 6). The study subjects who suffered from chronic illness and their attitude toward full protection against COVID-19 infection after getting a COVID-19 vaccine have a significant association. Different occupational categories have different attitudes toward future unforeseen problems due to the COVID-19 vaccine in individuals. HCWs that were willing to receive the COVID-19 vaccine, had different occupational categories, were suffering from chronic illnesses, and the attitude towards the COVID-19 vaccination program being a fraud had a significant association.

**Table 6:** Association of participants’ attitude association with demographic characteristics (multivariate analysis) (N=498) *P-value is significant at 0.05.

Attitude Statements	Demographic Characteristics													
	Age		Gender		Occupation		Tested positive for COVID-19		Family members tested positive for COVID-19		Suffering from chronic illness		Willing to receive COVID-19 vaccine	
	B	Sig	B	Sig	B	Sig	B	Sig	B	Sig	B	Sig	B	Sig
I feel that the COVID-19 vaccine is very safe	.066	.855	.505	.160	.349	.290	-.165	.662	.116	.806	-.158	.820	-2.10	<0.001*
I can rely on the COVID-19 vaccine to prevent serious infection with COVID- 19	-.104	.744	.316	.329	.090	.762	-.651	.082	.187	.670	-.878	.256	-.37	.354
I feel fully protected from COVID-19 infections in the future after getting the COVID-19 vaccine.	1.313	.364	14.59	.994	-15.10	.994	15.22	.994	1.768	.221	14.17	<0.001*	-14.79	.996
Although the COVID-19 vaccine appears to be safe, there may be problems that we haven’t yet discovered.	-.683	.057	-.463	.178	.141	.651	-.179	.615	.358	.379	-.848	.131	.188	.689
COVID-19 vaccine can cause unforeseen problems in individuals	-.061	.823	.296	.314	.888	<0.001*	-.076	.789	.083	.813	.265	.651	-.634	.167
I worry about the unknown effects of the COVID-19 vaccine in the future.	-.360	.225	.070	.816	.240	.357	-.372	.211	-.047	.891	-.056	.921	-1.53	.015*
COVID-19 vaccines will make a lot of money for pharmaceutical companies, but will not bring much benefit to common people.	-.116	.683	.017	.955	-.189	.462	.380	.206	.687	.053	.296	.557	-.137	.736
Authorities promote the COVID-19 vaccine for financial gain, not for people’s health.	.593	.105	.410	.230	-.452	.154	-.496	.212	.120	.782	1.454	.026*	-.372	.440
COVID-19 vaccination programs are a fraud.	-.011	.980	.716	.081	-1.45	.001*	.449	.310	-.610	.232	1.509	.019*	-1.40	.009*
Natural immunity will last longer than COVID-19 vaccination.	-.174	.527	-.228	.428	-.030	.904	-.100	.724	-.658	.068	.633	.284	-.539	.219
Natural exposure to viruses gives the safest protection against COVID-19.	.438	.105	-.030	.916	-.019	.939	-.079	.771	.266	.405	.780	.170	-.865	.063
Being exposed to COVID-19 naturally is safer for the immune system than being exposed through vaccination.	.256	.358	-.390	.188	-1.091	<0.001*	.087	.769	.579	.104	1.344	.050	-.531	.210

## Discussion

Healthcare workers with positive attitudes and practices toward vaccinations are more likely to recommend vaccinations to their patients. Patients often trust health care professionals for information about vaccines and vaccine-preventable diseases. This study acts as a guide for health ministries and public health experts in India for the maximum coverage of the COVID-19 vaccination.

In this study, 89.2% of participating health care workers were in favor of getting vaccinated with a COVID-19 vaccine, which is supported by another Asian study done by Chew N et al. [14]. Gagneux-Brunon A et al. also found that 75% of health care workers in France are willing to get vaccinated [17]. Our study is against a study done by Kabamba Nzaji M et al., who found that willingness to get vaccinated lies between 27.7% among health care workers in the Democratic Republic of Congo [18]. A study conducted in Greece by Kourlaba G et al. showed that almost half the population was hesitant about the SARS-CoV-2 vaccine [19].

We found that there is no gender gap in preferability to get vaccinated against COVID-19 as shown in other studies. Like Gagneux-Brunon A et al., Fu C et al. found that male health care professionals are more likely to accept COVID-19 vaccination than female HCWs [17,20-21]. Doctors and nurses had a higher acceptance of vaccination against COVID-19 than non-professionals like hospital attendants, office staff, and administration staff. The higher acceptance of COVID-19 vaccination among doctors and nurses may be due to direct contact with COVID patients, continued attendance of training classes and webinars, higher education levels, and following COVID-appropriate behaviors. Doctors and nurses had a more positive attitude toward vaccination than non-healthcare professionals like hospital attendants, administration staff, and clerical staff of the tertiary care institute. Non-healthcare professionals need information and education regarding vaccination safety and COVID-appropriate behavior. Participants who have chronic illnesses are less likely to receive the vaccination. They are worried regarding the possible side effects after getting a vaccination and show a negative attitude toward the COVID-19 vaccination.

The main reasons for the non-acceptability of vaccines among health care workers were insufficient knowledge of vaccine safety, concerns regarding future side effects after getting vaccinated, chronic illnesses, and absence of enough availability of safety and clinical trial data about COVID-19 vaccinations. The main factor that might increase vaccine acceptability among the participants is to provide sufficient and accurate information about the available vaccines, their trial data, and their possible side effects. We found that health care professionals are more confident regarding vaccination and less concerned about the side effects than non-healthcare professionals. One-hundred sixty-three health care workers believed that natural exposure to viruses gives the safest protection against COVID-19. The possible reason for this might be the younger age group of the study participants. The mean age of HCWs who participated in the study was 29.8 years. There are fewer chances of co-morbidity during these age groups, and they might be thinking that natural exposure is better than COVID-19 vaccination.

The majority of subjects (95.6%) had excellent knowledge as evidenced by the mean score of 7.17 ± SD 0.95 regarding COVID-19 and COVID-19-appropriate behavior. There was a significant correlation between getting the COVID-19 vaccine and the level of knowledge. The HCWs who had a higher level of knowledge, following COVID-19 appropriate behavior are more likely to receive the vaccination. Healthcare workers believed that they will wear masks, maintain social distance, and wear full PPE when dealing with COVID-19 patients after getting vaccinated too.

This study shows a neutral attitude toward the vaccination against COVID-19 but a number of studies in several countries have indicated negative attitudes toward vaccines against COVID-19 [22-25]. HCWs that are willing to receive a COVID-19 vaccine and their attitude toward the safety of COVID-19 vaccinations have a significant association. The neutral attitude of HCWs is the area of concern because if they are not confident enough regarding the COVID-19 vaccination and have a neutral attitude to COVID-19 vaccination then this will directly affect vaccine acceptance by the general population and that might be the long journey to cover the entire population for vaccination.

One-hundred forty-nine (29.9%) HCWs believed that being exposed to COVID-19 naturally is safer for the immune system than being exposed through vaccination but the majority of HCWs were in favor of vaccination against COVID-19. The occupation of different categories of HCWs had a significant correlation with attitude toward getting vaccinated against COVID-19. 195 respondents (39.2%) told that they believe natural immunity will last longer than the COVID-19 vaccination. One-hundred thirty (130; 26.1%) HCWs tested positive till the time of the study, and it is interesting to note that a respondent's previous experience with COVID-19 was not predictive of vaccine acceptance or hesitancy.

Strength and limitations of the study

The strength of this study is that in previous awareness and attitude studies, data collection was conducted using online self-reported questionnaires, which have the disadvantage of limiting the participation of vulnerable groups such as hospital attendants who are also major manpower of the health care system. This is the first study that involved all health care workers from doctors to the grassroots level such as hospital attendants and office staff of the hospital. In our survey, we used a sample with greater generalizability in terms of age (20-65 years) and occupation. We used the vaccine attitude scale, which was a standardized tool with effective validity and reliability. We also collected data to find out the reasons for the non-acceptability of the COVID-19 vaccine among health care workers, which makes the policymakers aware of their concerns and possible clarifications.

This study has limitations too. This study was done at the start of the vaccination campaign and was a cross-sectional study so it only shows the data of the health care workers' response at the point of the study. The actual intention of vaccination against COVID-19 could be different when the vaccine is widely distributed and accepted. In our study, 354 (71.1%) participants were male and only 28.9% were female so there was a gender gap. This study is limited to western India so the COVID-19 vaccination attitude may differ in other regions of the country.

Availability of data and materials

The datasets used and analyzed for the current study are available from the corresponding author on reasonable request.

## Conclusions

At the early stage of vaccination, vaccine acceptability among health care workers is higher, and they have excellent knowledge regarding COVID-19 and COVID-19-appropriate behavior. This will directly enhance the level and acceptability of vaccines among other health care workers of the country and the general population. The health care workers show a neutral attitude toward COVID-19 vaccination, which needs to be rectified by the government by providing health-related education. Appropriate vaccination strategies are essential for wider coverage of the population for vaccine uptake campaigns.
